# Biological Activities of Organic Extracts of the Genus *Aristolochia*: A Review from 2005 to 2021

**DOI:** 10.3390/molecules27123937

**Published:** 2022-06-20

**Authors:** Martín A. Lerma-Herrera, Lidia Beiza-Granados, Alejandra Ochoa-Zarzosa, Joel E. López-Meza, Pedro Navarro-Santos, Rafael Herrera-Bucio, Judit Aviña-Verduzco, Hugo A. García-Gutiérrez

**Affiliations:** 1 Instituto de Investigaciones Químico Biológicas, Universidad Michoacana de San Nicolás de Hidalgo, Ciudad Universitaria, Morelia 58030, Michoacán, Mexico; lidia.beiza@umich.mx (L.B.-G.); rafael.herrera.bucio@umich.mx (R.H.-B.); jaavina@umich.mx (J.A.-V.); 2 Centro Multidisciplinario de Estudios en Biotecnología, Facultad de Medicina Veterinaria y Zootecnia, Universidad Michoacana de San Nicolás de Hidalgo, Tarímbaro 58893, Michoacán, Mexico; ochoaz@umich.mx (A.O.-Z.); elmeza@umich.mx (J.E.L.-M.); 3 CONACYT—Universidad Michoacana de San Nicolás de Hidalgo, Edificio B-1, Ciudad Universitaria, Morelia 58030, Michoacán, Mexico; pedro.navarro@umich.mx

**Keywords:** *Aristolochia*, bioactivity, phytochemistry, ethnomedicinal

## Abstract

Different ethnomedicinal studies have investigated the relationship between various phytochemicals as well as organic extracts and their bioactive aspects. Studies on biological effects are attributed to secondary metabolites such as alkaloids, phenolic compounds, and terpenes. Since there have been no reviews in the literature on the traditional, phytochemical, and ethnomedicinal uses of the genus *Aristolochia* so far, this article systematically reviews 141 published studies that analyze the associations between secondary metabolites present in organic extracts and their beneficial effects. Most studies found associations between individual secondary metabolites and beneficial effects such as anticancer activity, antibacterial, antioxidant activity, snake anti-venom and anti-inflammatory activity. The aim of this review was to analyze studies carried out in the period 2005–2021 to update the existing knowledge on different species of the genus *Aristolochia* for ethnomedicinal uses, as well as pharmacological aspects and therapeutic uses.

## 1. Introduction

The Aristolochiaceae family is represented by seven genera: *Asarum*, *Saruma*, *Lactoris*, *Hydnora*, *Prosopanche*, *Thottea*, and *Aristolochia* [1]. About 550 species are known, distributed in the tropics and temperate zones of America, Asia, and Australia [2]. Traditionally, the Aristolochiaceae family was located in the Aristolochiales order by Cronquist (1981) and Takhtajan (1997). Recent studies indicate that it belongs to the Piperales order [3]. The genus *Aristolochia* is the most abundant of the Aristolochiaceae family and has been widely used in traditional Chinese medicine mainly [4], the genus is integrated by 550 species, making it the most important genus of the family [5]. Most of the species of this genus are perennial, herbaceous, distributed in bushes, in coiled or liana form, showy flowers, prostrate or tuberous rhizomes, as well as leaves with the presence of essential oils [6]. In the last two decades, the genus *Aristolochia* has generated great interest due to the abundance of mainly secondary metabolites, terpenes, and alkaloids [7,8,9].

*Aristolochias* species exist in various parts of the world; however, some species have been identified in Mexico: *A. buntingii* Pfeifer, *A. tresmariae* Ferris, *A. pacifica* Santana Mich. & Paizanni, *A. savannoidea* Paizanni & M. Ramírez, *A. tuitensis* Santana Mich. & Paizanni, *A. manantlanensis* Santana Mich., *A. malacophylla* Standl., *A. odoratissima* L., *A. styloglossa* Pfeifer, *A. foetida* Kunth, *A. tequilana* S. Watson, *A. luzmariana* Santana Mich. and *A. emiliae* Santana Mich. & Solís for which there are no phytochemical or biological studies showing the presence of active compounds [10,11,12]. Other species such as *A. cardiantha* Pfeifer, *A. flexuosa* Duch., *A. glossa* Pfeifer, *A. malacophylla* Standl., *A. mutabilis* Pfeifer, *A. mycteria* Pfeifer, and *A. tentaculata* O. C. Schmidt, have also been identified in the state of Michoacán, in localities near the Bajío area, in Mexico [13,14,15].

Some of the species of the genus *Aristolochia* are characterized by having compounds such as aristolochic acids that are attributed to adverse health effects. However, these compounds can be related to other lower-risk applications. Otherwise, there are also phenolic and terpene compounds that show beneficial effects in different biological aspects, which is why it is important to know which ones are related to the different species for subsequent studies. Therefore, this systematic review examined the published pharmacological and ethnomedicinal literature of different *Aristolochias* species for possible studies associated with phytochemicals from organic extracts and beneficial effects.

## 2. Beneficial Effects of *Aristolochia* Genus

The secondary metabolites responsible for the biological effects of the species of the *Aristolochia* genus generally are usually aristolochic acids and their derivatives, as well as monoterpenes such as thujene, camphene, and carene, kaurene-type diterpenes, triterpenes such as lupeol, among others. Likewise, alkaloid metabolites derived from aristolactams and phenolic compounds of the lignan type are involved in these functions [9,16,17,18].

*Aristolochia* is the most abundant genus in the Aristolochiaceae family. The species of this genus are used ornamentally and in traditional medicine as a source of abortifacients, emmenagogues, sedatives, analgesics, anti-cancers, anti-inflammatories, muscle relaxants, antihistamines, antiparasitics, to treat cholera, abdominal pain, rheumatism, antimalarial, skin problems, and different types of bites and stings from animals and insects [9].

The use of plant extracts in traditional medicine is profitable because no elaborate procedures are required to obtain them, production costs are low, and the materials to obtain them are accessible [19,20]. For these reasons, several studies have used extracts of different solvents to obtain metabolites using different parts of the plant. The extracts as well as the active compounds that comprise the *Aristolochia* species have been used in pharmacological aspects and in traditional medicine frequently in recent years.

### 2.1. Ethnomedicinal Use

A variety of traditional uses for species of the genus *Aristolochia* were found in the literature. Of the traditional uses cited, the most common uses are anticancer (33 articles) [21,22,23,24,25,26,27,28,29,30,31,32,33,34,35,36,37,38,39,40,41,42,43,44,45,46,47,48,49,50,51,52,53], antibacterial (31 articles) [23,25,29,30,38,39,45,49,54,55,56,57,58,59,60,61,62,63,64,65,66,67,68,69,70,71,72,73,74,75,76], antioxidants (18 articles) [22,25,29,33,34,46,55,60,61,70,73,74,77,78,79,80,81,82], snake anti-venom (13 articles) [21,22,39,40,76,83,84,85,86,87,88,89,90], anti-inflammatory (11 articles) [22,40,46,47,74,86,91,92,93,94,95], abdominal pain (11 articles) [17,21,22,23,39,40,48,76,96,97,98], antiparasitic (7 articles) [18,39,75,83,99,100,101], insecticide an predator protection (7 articles) [40,102,103,104,105,106,107], anti-malarial (5 articles) [21,40,48,108,109], skin diseases (5 articles) [22,23,40,76,86], fever (4 articles) [7,21,22,48], headache (4 articles) [21,22,48,85]. Other beneficial effects such as, antifungal activities [45,62,110], antinociceptive [94,111,112], changes in the estrous cycle [113,114], antifibrosis [115,116], hepatoprotection, nephroprotection [117], neuroprotective effect [118], antiurcer [119], antiallergic [120], immune effect [121], angiogenic [122], osteogenic differentiation of gingival mesenchymal stem cells [123], antidiabetic [22,124,125], control of melanogenesis [126], antihemorrhagic [127], antispasmodic [97], antitoxin [128], liver protector [100], bronchitis, constipation, rheumatism and bladder diseases [129], heart protector [130], antidyslipidemic [82], healing of wounds [98], acaricide [131], expectorant, antitussive, antihistamine and pain reliever [89].

Also, traditional uses include mainly the root of the plant (42 articles), the leaves (31 articles), the stems (17 articles), aerial parts (15 articles), and the whole plant (15 articles). Some forms of use of *Aristolochia* plants for ethnomedicinal use in snakebites are drinking whole plant juice and leaves, aqueous extract (AE) orally and applying a root paste to the wound and giving a root paste orally. In skin diseases, the shade-dried root powder is taken orally for 48 days. In fever, the leaves are chewed during the illness. The headache is treated with the formation of a paste placed on the forehead. In abdominal pain, the use of a decoction of the roots is used. In the treatment of malaria, the plant is used in decoction [21,22,108].

### 2.2. Phytochemical Studies

The review of the literature allowed knowing phytochemicals that have a higher prevalence such as phenanthrene derivatives, phenolic compounds, fatty acids, and isoprenoid derivatives. Extracted and polar roots showed a higher prevalence of phenanthrene derivatives and phenolic compounds. The roots and aerial parts of the medium and low-polarity extracts showed a higher presence of fatty acids and derived isoprenoids. The most prominent phytochemicals are shown in Table 1.

### 2.3. Pharmacological Activity

Pharmacological studies have been carried out using crude extracts and bioactive compounds from different species of *Aristolochia*. The beneficial effects that most prevailed in this review were: anticancer activity, antibacterial, antiparasitic and antiviral activity, antiplatelet activity, antioxidant activity, neuroprotective activity, changes in the estrous cycle, antidiabetic potential, anti-inflammatory activity, and antifibrotic activity. Table 2 shows the common beneficial and ethnomedicinal effects of *Aristolochia* species in traditional medicine.

#### 2.3.1. Anticancer Activity

In aerial parts of *A. longa* L., a greater in vitro cytotoxic effect was determined on RD (embryonal rhabdomyosarcoma cells) (IC_50_ = 0.015 mg/mL) of a dichloromethane extract (DCME), followed by the hexane extract (HXE) on BSR (kidney adenocarcinoma of hamster cells) (IC_50_ = 0.018 mg/mL). The least cytotoxic effect was shown in the HXE and DCME analyzed in Vero (monkey kidney cancer cells) cells (IC_50_ = 0.250 mg/mL) as well as in the methanolic extract (ME) of RD (IC_50_ = 0.200 mg/mL) and BSR (IC_50_ = 0.350 mg/mL). The compounds implicated in this beneficial activity are attributed to linoleic acid chloride, oleic acid, and limonene-6-ol, pivalate [23]. The possible mechanisms of cytotoxicity of the compounds characterized in the HXE and DCME could be related to the cleavage of the plasma membrane and the release of its content into the extracellular medium [24]. *A. longa* L. exhibited an in vitro cytotoxic effect of HXE of the root on RD cells (IC_50_ = 0.0151 mg/mL) showing a relationship of its activity to flavonoids (76.41 ± 8.74 mg GAE/g), while the HXE the cytotoxicity in healthy PBMC (human peripheral blood mononuclear) cells was lower (IC_50_ = 0.0625 mg/mL) [25]. The chloroform extract (CE) from the roots of *A. baetica* L. showed cytotoxic activity (IC_50_ = 0.2160 mg/mL) in vitro against MCF-7 (breast cancer cells) by means of the MTT [3-(4,5-dimethylthiazol-2-yl)-2,5-diphenyltetrazolium bromide] colorimetric assay. Aristolochic acid I was identified and contributed to the cytotoxicity of the extract [26].

A study of the CE of leaves of *A. indica* L. was carried out and cytotoxicity was obtained with the MTT assay at 48 h after treatment in MCF-7 cells (IC_50_ = 0.347 mg/mL) using Taxol™ (IC_50_ = 1.17 × 10^−8^ M) as a standard control. The compounds identified in the CE of the leaves were flavonoids, tannins, glycosides, phenols, saponins, terpenoids, and amino acids [27].

Compounds such as alkaloids, flavonoids, steroids, and anthraquinones from the aerial parts of the CE of *A. ringens* Vahl. caused a cytotoxic effect against HepG-2 (human liver cancer cells) (IC_50_ = 0.0164 mg/mL) and on MCF-7 cells (IC_50_ = 0.0816 mg/mL) [28].

In DCME collected in 2018 from *A. foetida* Kunth, IC_50_ values were determined for leaves of 0.0473 mg/mL and for stems with IC_50_ values of 0.0459 mg/mL in MCF-7 cells. Components in the extracts can cause late apoptotic cell death through the intrinsic pathway in the cancer cell line. The main compounds identified were methyl hexadecanoate; hexadecanoic acid; 2-butoxyethyl dodecanoate; ethyl hexadecanoate; methyl octadeca-9,12,15-trienoate; and (9*Z*,12*Z*,15*Z*)-octadeca-9,12,15-trienoic acid that allow cytotoxic activity [24].

Essential oils from the aerial parts of *A. fordiana* Hemsl. were evaluated against HepG-2 cells (IC_50_ = 0.69 mg/mL) and the MCF-7 cell line (IC_50_ = 0.22 mg/mL) for 72 h attributing its effect to the compounds *β*-caryophyllene, limonene, and linalool. Doxorubicin was used as a positive control in HepG-2 (IC_50_ = 0.00049 mg/mL) and MCF-7 (IC_50_ = 0.00022 mg/mL) [29]. The sesquiterpene 2,2,7,7-tetramethyltricyclo[6.2.1.0(1,6)]undec-4-en-3-one has been identified and characterized as the main compound in essential oils of *A. mollissima* Hance. Essential oils from rhizomes showed cytotoxic activity in ACHN (kidney adenocarcinoma cells) (IC_50_ = 0.0223 mg/mL), MCF-7 (IC_50_ = 0.0206 mg/mL), Bel-7402 (human liver carcinoma cells) (IC_50_ = 0.0331 mg/mL), HepG-2 (IC_50_ = 0.0332 mg/mL), and HeLa (human cervix carcinoma cells) (IC_50_ = 0.0386 mg/mL) compared to aerial parts with the exception of MDA-MB-435S (melanoma cells) (IC_50_ = 0.0203 mg/mL) [30].

The cytotoxic effect of an AE of the root of *A. longa* L. on breast cancer cell lines was evaluated in vitro by means of the MTT assay, whose activity may be related to flavonols, flavones, and/or flavonoid glycosides [50]. On the other hand, tests were carried out on human red blood cells with an AE of aerial parts of *A. longa* L. collected in Algeria in March 2018. The AE did not show high percentages of hemolysis (68.75 ± 6.11%; 200 mg/mL). The concentration of polyphenols [283.68 ± 0.60 mg GAE (gallic acid equivalent)/g] and flavonoids (10.50 ± 0.03 mg QE (quercetin equivalent)/g) could influence hemolysis, which is important to consider the dose of the AE in traditional cancer medicine [51].

Ethanol extract (EE) and DCME:ME from *A. ringens* Vahl. roots were evaluated in vitro and in vivo and compared with 5-fluorouracil. However, the study lacked the characterization of the bioactive compounds to corroborate their anticancer therapeutic approach [31]. Likewise, in the ME of leaves of the species *A. macroura* Gomes., the active components were not specifically mentioned and their cytotoxic activity against HepG-2 cells (IC_50_ = 0.513 mg/mL) was higher compared to other species such as *Schinus molle* L. (IC_50_ = 0.050 mg/mL) [32].

In a chemical and biological study of *A. maurorum* L., the main components of roots and aerial parts of ME were aristolochic acid I, II, and IIIa. However, the compound that showed the greatest cytotoxic effect was aristolochic acid I (IC_50_ = 1.43 × 10^−8^ M, in *Artemia salina*); it is worth mentioning that the biological evaluation of the cytotoxic activity was not carried out in cancer cells [53]. The ME of the roots of *A. baetica* L. demonstrated antiproliferative effect against T-24 (human bladder cancer cells) IC_50_ = 0.048 mg/mL and HT-29 (human colon cancer cells) IC_50_ = 0.100 mg/mL relative to HepG-2 (IC_50_ = 0.380 mg/mL). The antiproliferative effect can be attributed to phytochemicals identified mostly as polyphenols, alkaloids, flavonoids, saponins, and tannins and their possible mechanism of action against cancer cells via intrinsic apoptosis [33]. The polar extracts such as the ME ones mentioned above, as well as the EE one from the roots of *A. bracteolata* Lam. have shown highly effective cytotoxic activity against MCF-7 cells (IC_50_ = 0.0191 mg/mL), where saponins, alkaloids, flavonoids, sterols, and carbohydrates were identified as major components [34]. The mechanism of cell death against cancer cells that phenolic compounds can present involves the inhibition of enzymes compromising the cell cycle [132]. The ME of stems and leaves of *A. tadungensis* T. V. Do & Luu. was evaluated in HeLa (IC_50_ = 0.0083 mg/mL), PANC-1 (human pancreas cell line) IC_50_ = 0.0826 mg/mL, and A-549 (human lung cell line) IC_50_ = 0.0755 mg/mL. The aristolochiaside compounds with cytotoxic effect on HeLa (IC_50_ = 7.59 × 10^−6^ M) and on PANC-1 (IC_50_ = 5.47 × 10^−5^ M) were characterized and identified. Only in the PANC-1 cell line the IC_50_ values were > 2.5 × 10^−5^ M [133]. Aristolactam AIIIa showed cytotoxicity against A-549 cells (IC_50_ = 2.40 × 10^−5^ M). Camptothecin (1.35 × 10^−6^ M) was used as a control [35]. Aristolactam AIIIa can induce apoptosis and cell cycle arrest in the G2/M phase in cancer cells [134]. In particular, in the EE of the rhizomes of *A. championii* Merr. & Chun. The aristolochic acid derivative aristchamic-A showed higher cytotoxic activity against HCT-116 (human colon cancer cells) IC_50_ = 5.00 × 10^−7^ M, HepG-2 (IC_50_ = 7.37 × 10^−6^ M), BGC-823 (human gastric carcinoma cells) IC_50_ = 2.66 × 10^−6^ M and NCI-H1650 (human lung cancer cell line) IC_50_ = 7.50 × 10^−7^ M. The activity of aristolochic acid derivatives could be associated with the 9,10-dihydroaristolochic acid skeleton [36]. From an EE of roots, aristolochic acid I was identified in *A. indica* L., which showed antitumor action in adenocarcinoma 755 in mice at a dose of 2 mg/kg [22]. At low doses, aristolochic acids can arrest the G2/M phase of the cell cycle and cause DNA damage by increasing reactive oxygen species (4.0 × 10^−6^ M) as well as activating apoptosis in higher doses (4.0 × 10^−5^ M) [135]. Despite the controversy over the nephrotoxicity and carcinogenic effects of aristolochic acids and their derivatives, they can be focused on cytotoxic treatments [136].

The cytotoxic effect on MG-63 (human osteosarcoma cells) was determined with eupomatenoid-7 (IC_50_ = 1.19 × 10^−5^ M) and HepG-2 with eupomatenoid-5 (IC_50_ = 9.15 × 10^−6^ M) isolated from the EE of aerial parts of *A. fordiana* Hemsl. Cisplatin was used as a positive control against MG-63 (IC_50_ = 5.31 × 10^−6^ M) and HepG-2 (IC_50_ = 5.21 × 10^−6^ M) [37].

On the other hand, in the species *A. galeata* Mart., a cytotoxic effect was found against HeLa cells of the ethanolic extract (IC_50_ = 0.369 mg/mL) and by partitioning the dichloromethane fraction (IC_50_ = 0.09 mg/mL) was obtained whose cytotoxic effect was greater with respect to the fractions of hexane, ethyl acetate, and hydroethanolic. The secondary metabolites determined in the EE and the dichloromethane fraction were flavonoids, steroids, and triterpenes [38].

In HK-2 (renal cells), 28 ME from different species of the genus *Aristolochia* were tested, so that aristolactam BI, aristolochic acid D, and aristolactam IIIa may be responsible for the genotoxic and cytotoxic activity. The possible mechanism of action of aristolochic acids and their derivatives causes apoptosis and arrest of the G2/M phase of the cell cycle [137]. Of the 68 extracts tested on cancer cells, 31 extracts had an IC_50_ < 0.1 mg/mL [133]. Table 3 shows different cancer cell lines against organic extracts of different species of the genus *Aristolochia*.

#### 2.3.2. Antibacterial, Antiparasitic and Antiviral Activity

Mohanraj et al. (2009) identified from essential oils of leaves of *A. elegans* Mast. sesquiterpenes β-caryophyllene and *iso*-caryophyllene with antibacterial activity against *Klebsiella pneumoniae, Vibrio cholerae, Salmonella typhi*, and *S*. *paratyphi* A. The aforementioned compounds, as well as bicyclogermacrene, are attributed to antiviral activity against the HIV-1 antigen p24 with an inhibition of 35.6–14.9% [54]. Phenolic compounds such as fargesin, (8*R*,8′*R*,9*R*)-cubebin and eupomatenoid-1 were identified in HXE from the rhizomes of *A. elegans* Mast. which favored the inhibition of *M. tuberculosis* at a minimum inhibitory concentration (MIC) of 50 µg/mL. Eupomatenoid-1 showed antiparasitic activity (IC_50_ < 1.93 × 10^−9^ M) against *E. histolytica* and *G. lamblia* [39]. Navarro-García et al. (2011) determined that in the DCME from *A. brevipes* Benth. roots collected in Mexico, the aristolactam I presented greater antibacterial activity against *Mycobacterium tuberculosis* H37Rv with an MIC between 8.52 × 10^−8^ and 4.26 × 10^−8^ M [76]. Likewise, in *A. taliscana* Hook. & Arn., the rhizome HXE exhibited antibacterial activity (MIC = 0.7 mg/mL) as well as the isolated compound eupomatenoid-7 (MIC = 2.15 × 10^−6^ M) inhibiting the growth of *Escherichia coli, Pseudomona fluorescens*, and *Listeria monocytogenesis* [55]. In the research carried out by León-Díaz et al. (2013), the HXE root of *A. taliscana* Hook. & Arn. (−)-licarin-A was isolated whose concentration of 5 mg/kg reduced pneumonia in mice infected with *M. tuberculosis* [56]. The linoleic acid chloride, oleic acid, and limonene-6-ol, pivalate were isolated from DCME from the tubers of the *A. longa* L. species, the present activity was evident against *Rhodococcus* sp: *R. equi,* GK1, and GK3 (with an inhibition zone of 30 mm at 50 mg/mL) [23]. The HXE of *A. longa* L. exhibited antibacterial activity (10 mg/mL) against *Staphylococcus aureus*, determining a total inhibitory effect with a zone of inhibition of 8.5 mm. The antibacterial activity may be related to the amount of polyphenols and flavonoids in the organic extract of *A. longa* L. [25]. Essential oils promote the loss of the integrity of the cell membrane by releasing the cell material to the external environment, in addition to the inhibition of proteins and biofilms [138]. It is worth mentioning that the extracts of *A. longa* L. mentioned above exceed 0.1 mg/mL, so they would not be suitable for use as antibacterials [133].

#### 2.3.3. Antiplatelet Activity

In *A. maurorum* L., the main components of the roots and aerial parts of the ME were aristolochic acid I (1.17 × 10^−6^ M), II (1.28 × 10^−6^ M), and IIIa (1.22 × 10^−6^ M). These components showed an antiplatelet activity of 100% and the assay was compared with the standard acetylsalicylic acid (3.05 × 10^−5^ M) showing an inhibition of platelet aggregation of 100%. Compounds were evaluated using an automatic platelet aggregometer and coagulation tracer [53].

#### 2.3.4. Antioxidant Activity

In *A. taliscana* Hook. & Arn. in HXE of rhizomes, the ABTS assay (2,2′-azino-bis(3-ethylbenzothiazoline-6-sulfonic acid) was performed to measure the ability of the compounds to trap the ABTS^•+^ radical. The results obtained were expressed as antioxidant activity of eupomatenoid-7 (151.2 mg GAE/g) and (±)-licarin-A (143.4 mg GAE/g) and were the most active at both points of the determination (minute 1 and 7 of the reaction) [55]. The antioxidant activity is dependent on hydroxyl groups, to which its antioxidant effect is attributed, which is why licarin-B and eupomatenoid-1 did not present this condition.

*A. bracteolata* Lam. showed activity to chelate iron with an antioxidant capacity of 44 ± 0.01%, whose activity is attributed to phenolic compounds [34]. In the ME of *A. longa* L., it was determined that it has a high amount of polyphenols and flavonoids, and it showed a remarkable antioxidant activity. The total content of phenolic compounds of *A. longa* L. showed that the ME of roots presented the concentrations of polyphenols and flavonoids with 101.4 mg of GAE/g and 54.21 mg of QE/g of extract, respectively [25].

#### 2.3.5. Neuroprotective Activity

Dihydrobenzofuran neolignans, 2-aryldihydrobenzofurans, 8-*O*-4′-neolignan, and analogs (3.0 × 10^−5^ M) as well as the EE of the stem (0.01 mg/mL) of *A. fordiana* Hemsl. exhibited a neuroprotective effect that prevents cell death in hippocampal cells (HT-22) [118].

### 2.4. In Vivo Studies on Extracts of the Genus Aristolochia

#### 2.4.1. Changes in the Estrous Cycle

In tubers of the EE of the species *A. indica* L., an application has been found regarding changes in the estrous cycle in vivo with a dose of 150 mg/kg of extract [113]. The compounds involved in the effect of the extract were not shown.

#### 2.4.2. Antidiabetic Potential

From the EE of roots of *A. ringens* Vahl., aristolone was identified and it was shown to have an antidiabetic potential in rats at concentrations of 300–75 mg/kg, so this part of the plant could be used in decoctions for the treatment of diabetes with the approval of more relevant studies [125].

#### 2.4.3. Antifibrotic Activity

The compounds (+)-*iso*-bicyclogermacrenal and spatulenol (3.0 × 10^−5^ M) present in the ethyl acetate extract (EAE) of *A. yunnanensis* Franch. stems were responsible for promoting antifibrotic concentration effects in vivo [116]. However, the concentrations in which the pure compound was handled under in vivo conditions turned out to be high for antifibrotic activity. The genus *Aristolochia* has extensive traditional and pharmacological uses in various pathological conditions. Therefore, it is an attractive subject for future clinical and experimental research.

#### 2.4.4. Anti-Inflammatory Activity

In particular, in *A. krisagathra* Sivar. & Pradeep., studies of EE of the whole plant have been carried out. An anti-inflammatory activity of 87.1% was obtained with a dose of 400 mg/kg in rats. The compounds that could act in biological activity are alkaloid, anthraquinone, coumarin, flavonoid, phenol, quinone, saponin, steroid, tannin, terpenoid, sugar, glycoside, and xanthoprotein [95].

The anti-inflammatory activity of (−)-hinokinin in tumor necrosis factor-*α* (TNF-*α*) IC_50_ = 0.0775 M and interleukin-6 (IL-6) IC_50_ = 0.0205 M and aristolactam I (TNF-*α*; IC_50_ = 0.1168 M, IL-6; IC_50_ = 0.0520 M) of *A. indica* L. in aerial parts of the DCME and EAE, respectively [22]. In in vivo and in vitro studies, doses greater than 200 mg/kg are not usually recommended, as well as values in pure compounds > 2.5 × 10^−5^ M [133,139].

#### 2.4.5. Snake Anti-Venom Activity

The hexanic extract from the roots of *A. elegans* Mast. was subjected to an inhibition assay of smooth muscle contraction induced by scorpion venom (*Centruroides limpidus limpidus*) in an isolated guinea pig ileum model with an inhibition of 41.66% (0.4 mg/mL), whose effects are related to neolignan-type compounds [84]. On the other hand, in vivo studies in albino mice using a ME from the whole plant of the species *A. indica* L. demonstrated neutralization against *Daboia russelli* venom at a dose of 0.14 mg. However, no mention is made of the metabolites responsible for the activity [90].

Compounds obtained from polar extracts, especially aristolochic acids, as mentioned above, are not considered safe compounds according to the International Agency for Research on Cancer (WHO), due to their carcinogenic effects. Despite developing these problems, they can be oriented towards their possible use as antivenoms. Likewise, the presence of aristolochic acids, aristolactams, and their derivatives can be used as chemotaxonomic markers in species of the genus *Aristolochia* [22,136,140].

#### 2.4.6. Cancer Treatment

The AE of *A. longa* L. roots (5000 mg/kg) did not show hepatic and renal toxicity in a preclinical assay by oral administration in rats. More studies are warranted on its possible use in breast cancer therapy. The possible compounds responsible for the beneficial activity could be the flavonols, flavones, and/or flavonoid glycosides identified in the extract [50]. In addition to the bioactive compounds mentioned above, the amount of lectin in *A. longa* L. extracts was not favorable for potential cancer treatment in an in vitro immunological activity assay [141]. The use of AE of *A. longa* L. rhizomes as in vivo anticancer treatment in gingival tumorigenesis caused tissue damage as well as pulmonary and toxicity problems. This could be due to the presence of aristolochic acids in the extract [52]. In a preclinical assay against S-180 solid tumors from BALB/c mice, *A. ringens* Vahl. roots from extracts of EE (120 mg/kg) and DCME:ME (110 mg/kg) produced a significant value (*p* < 0.05) in tumor growth over a period of 9–13 days compared to control models. However, the characterization of the polar and moderately polar extracts lacked phytochemical information [31].

## 3. Materials and Methods

A total of 141 publications were included in this review. SciFinder and EBSCO were used to search for articles that analyzed the beneficial effects of *Aristolochia* in the period from 2005 to 2021. Eighty-eight different species of *Aristolochia* were considered and reviewed by International Plant Names Index and World Flora Online. The inclusion criteria that were retained included: phytochemicals, *Aristolochia*, beneficial effects, extract, pharmacology, and ethnomedicinal. Articles were excluded based on the following criteria: articles that did not address the intervention, articles without adequate *Aristolochia* species theoretical foundations, and articles that did not include *Aristolochia* species.

## 4. Conclusions

The review in the literature about biological activities allowed identifying studies of different species of the genus *Aristolochia* highlighting phytochemical and pharmacological aspects, and their possible clinical applications. In the roots and leaves, a greater number of beneficial effects were found. From this review, it is concluded that the information detailed the relevant species of the genus *Aristolochia* as promising candidates for natural uses in human health of greater relevance in extracts and pure compounds in anticancer activities. More selective studies are suggested in terms of concentration parameters as well as clinical studies for higher quality.

## Figures and Tables

**Table 1:** Main phytochemicals of species of the genus *Aristolochia*, using different solvents.

Phytochemicals	Species	Plant Part ^1^	Extract/Solvent	References
Polyphenols, alkaloids, flavonoids, saponins, tannins	*A. baetica* L.	RT	ME	[33]
Aristolochic acid I	*A. baetica* L.	RT	CE	[26]
Saponins, alkaloids, flavonoids, sterols, carbohydrates	*A. bracteolata* Lam.	RT	EE	[34]
Aristolactam I	*A. brevipes* Benth.	RT	DCME	[76]
Aristchamic-A	*A. championii* Merr. & Chun.	RZ	EE	[36]
*β*-caryophyllene, *iso*-caryophyllene, Bicyclogermacrene	*A. elegans* Mast.	LV	N/A	[54]
Fargesin, (8*R*,8′*R*,9*R*)-cubebin, eupomatenoid-1	*A. elegans* Mast.	RZ	HXE	[39]
Methylhexadecanoate; hexadecanoic acid; 2-butoxyethyl dodecanoate; ethylhexadecanoate; methyl octadeca-9,12,15-trienoate, (9*Z*,12*Z*,15*Z*)-octadeca-9,12,15-trienoic acid	*A. foetida* Kunth.	LV, S	DCME	[24]
*β*-caryophyllene, limonene, linalool	*A. fordiana* Hemsl.	AP	Et_2_O	[29]
Benzofuranneolignans, (−)-licarin-B, parakmerin A, perseal G, (+)-conocarpan, (7*R*,8*R*)-3,4-methylenedioxy-4′,7-epoxy-8,3′-neolignan-7′- [*E*]-ene, (+)-*trans*-dehydrodiisoeugenol, decurrenal, (2*R*,3*R*)-2,3-dihydro-2-(4-hydroxyphenyl)-7- methoxy-3-methyl-5-(*E*)-propenylbenzofuran, eupomatenoid-13, eupomatenoid-7, eupomatenoid-6, eupomatenoid-5	*A. fordiana* Hemsl.	AP	EE	[37]
Dihydrobenzofuran neolignans, 2-aryldihydrobenzofurans, 8-*O*-4′-neolignan and analogs	*A. fordiana* Hemsl.	S	EE	[118]
Flavonoids, steroids, and triterpenes	*A. galeata* Mart.	RZ	EE	[38]
Aristolic acid	*A. indica* L.	RT	CE	[22]
Aristolochic acid I	*A. indica* L.	RT	EE	
Aristolochicacid II	*A. indica* L.	LV	ME	
Aristolochicacid D	*A. indica* L.	RT	ME	
Aristololactam-I *N*-*β*-*D*-glucoside	*A. indica* L.	RT	Et_2_O	
(12*S*)-7,12-secoishwaran-12-ol	*A. indica* L.	RT	Et_2_O	
*β*-sitosterol	*A. indica* L.	RT	EE	
(−)-hinokinin	*A. indica* L.	AP	DCME	
Aristolactam I	*A. indica* L.	AP	EAE	
*β*-caryophyllene and *α*-humulene	*A. indica* L.	AP	N/A	[114]
Flavonoids, tannins, glycosides, phenol, saponins, terpenoids, amino acid	*A. indica* L.	LV	CE	[27]
Alkaloid, anthraquinone, coumarin, flavonoid, phenol, quinone, saponin, steroid, tannin, terpenoid, sugar, glycoside, xanthoprotein	*A. krisagathra* Sivar. & Pradeep.	WP	EE	[95]
Linoleic acid chloride	*A. longa* L.	AP	HXE	[23]
Oleic acid	*A. longa* L.	AP	HXE	
Limonene-6-ol, pivalate	*A. longa* L.	AP	HXE	
Starch, tannins	*A. longa* L.	RT	H_2_O	[25]
Tannins, flavonoids, coumarins, anthocyans	*A. longa* L.	RT	ME	
Polyphenols, flavonoids	*A. longa* L.	RT	HXE	
Flavonols, flavones, and/or flavonoid glycosides	*A. longa* L.	RT	H_2_O	[50]
Polyphenols, flavonoids	*A. longa* L.	RT	H_2_O	[51]
Aristolochic acid I	*A. maurorum* L.	RT	ME	[53]
Aristolochic acid II	*A. maurorum* L.	RT	ME	
Aristolochic acid IIIa	*A. maurorum* L.	RT	ME	
2,2,7,7-tetramethyltricyclo [6.2.1.0(1,6)]undec-4-en-3-one, (*E*)-*β*-santalolacetate, camphene, spathulenol, *β*-caryophyllene, *α*-humulene	*A. mollissima Hance.*	RZ	N/A	[30]
Alkaloids, flavonoids, steroids, anthraquinones	*A. ringens* Vahl.	AP	CE	[28]
Aristolochiaside, aristolactam AIIIa	*A. tadungensis* T. V. Do & Luu.	S, LV	ME	[35]
(±)-licarin-A and -B, eupomatenoid-1 and -7	*A. taliscana* Hook. & Arn.	RZ	HXE	[55]
(−)-licarin-A	*A. taliscana* Hook. & Arn.	RT	HXE	[56]
(+)-*iso*-bicyclogermacrenal	*A. yunnanensis* Franch.	S	EAE	[116]
Spatulenol	*A. yunnanensis* Franch.	S	EAE	

^1^ AP = aerial parts, LV = leaves, RT = roots, RZ = rhizomes, S = stems, WP = whole plant. N/A = not applicable. CE = chloroformic extract, DCME = dichloromethane extract, EAE = ethyl acetate extract, EE = ethanol extract, HXE = hexanic extract, ME = methanol extract, Et_2_O = ether.

**Table 2:** Ethnomedicinal uses and biological activities of *Aristolochia* species.

Species	Plant Part ^1^	Beneficial Effects	References
*A. acuminata* Lam.	FT, LV, RT, and S	Abdominal pain, abortifacient, analeptic, antipyretic, anti-inflammatory, bone fracture, bilious disorders, carminative, diarrhea, dysentery, emmenagogue, health tonic, loss of appetite, antimalarial, muscle relaxant, rheumatism, regulate menstrual disorders, snake bite, stomachache, swollen limbs, stimulate uterine flow, snake and scorpion poison, tumor, venereal disease	[40]
*A. albida* Duch.	RT	Larvicide, antiparasitic, snake antivenom	[83]
*A. arcuata* Mast.	LV	Hepatoprotection, nephroprotection	[117]
	LV	Protection against insects	[102]
*A. argentina* Griseb.	WP	Antimicrobial	[57]
	WP	Antiseptic, diuretic, emmenagogue, antioxidant	[77]
	AP	Insecticide	[103]
*A. baetica* L.	RT	Antioxidant, antiproliferative	[33]
	RT and LV	Antiproliferative	[26]
*A. birostris* Duch.	AP	Antimicrobial	[58]
*A. bracteata* Retz.	RT	Antimicrobial	[59]
	WP	Antiulcer	[119]
	WP and RT	Antioxidant	[60,78]
*A. bracteolata* Lam.	WP	Antiallergic	[120]
	FT, LV and RT	Insecticide	[104]
	WP	Antioxidant, antimicrobial	[73]
	AP	Anti-inflammatory	[91]
	LV	Immune effect	[121]
	AP	Angiogenic	[122]
	AP	Osteogenic differentiation of gingival mesenchymal stem cells	[123]
	LV	Antidiabetic	[124]
	RT	Cytotoxic, antioxidant	[34]
	AP	Control of melanogenesis	[126]
	WP, RT and LV	Gastric stimulant treatment, cancer treatment, lungs inflammation dysentery, and snake bite, treatment of malaria, convulsions, abdominal pain, scorpion stings, flu, vomiting, pneumonia, polymenorrhea and edema, fever, headache, general body pain, stomachache, diarrhea, and flu	[21]
*A. brevipes* Benth.	RZ	Antimycobacterial, antidiarrheal, arthritis, wound cleaner, and snake antivenom	[76]
	RZ	Antimycobacterial	[60]
*A. bodamae* Dingler.	RT	Antibacterial, antioxidant	[61]
*A. cathcartii* Hook.	LV, RZ, RT, and S	Food poisoning, insect repellent, liver disorders, promotes flow of urine, stomach ailments	[40]
*A. championii* Merr. & Chun.	RZ	Cytotoxic	[36]
*A. chilensis* Bridges ex Lindl.	S and LV	Antihemorrhagic	[127]
*A. clematitis* L.	RZ	Antibacterial, antifungal	[62]
	AP	Antioxidant	[79]
*A. constricta* Griseb.	AP	Antispasmodic	[97]
*A. cordigera* Willd. Ex Klotzsch.	S, LV, and RT	Antiprotozoal	[99]
*A. cymbifera* Mart.	LV, RT	Antitrypanosomal, antischistosomal	[18]
*A. debilis* Siebold & Zucc.	RT	Anti-inflammatory	[92]
	RT	Cytotoxic	[41]
*A. delavayi* Franch.	AP	Antibacterial	[63]
*A. elegans* Mast.	RZ	Antiparasitic and antimycobacterial, antibacterial, antitumor, antidiarrheal, antipyretic, snake bites	[39]
	RT	Antitoxin	[128]
	LV	Antifungal	[110]
	LV	Antiviral, antibacterial	[54]
	RT	Scorpion antivenom	[84]
*A. esperanzae* Kuntze.	RT	Antibacterial	[64,65]
*A. fangchi* Y. C. Wu ex L. D. Chou & S. M. Hwang.	RT	Cytotoxic	[42]
*A. foetida* Kunth.	WP	Snake bite, headache	[85]
	RT	Fever, colds, chills, asthma treatment	[7]
	LV and S	Cytotoxic	[24]
*A. fordiana* Hemsl.	WP	Cytotoxic	[37]
	WP	Antibacterial, cytotoxic and antioxidant	[29]
	S	Neuroprotective effect	[118]
*A. galeata* Mart.	RZ	Antibacterial and cytotoxic	[38]
*A. gehrtii* Hoehne.	LV	Liver protector and antiparasitic	[100]
*A. griffithii* Hook.f. & Thomson ex Duch.	RT	Antimalarial	[108]
*A. gigantea* Mart.	RT	Antitrypanosomal	[75]
*A. indica* L.	RT	Fertility regulator	[114]
	RT	Antidiarrheal	[17]
	RT	Cytotoxic	[43]
	LV	Antibacterial	[66]
	S and LV	Antibacterial	[67]
	LV	Anti-inflammatory, poisonous bites, gastric stimulator, skin problems, antidiarrheal, antipyretic, antitussive	[86]
	LV	Snake bites	[87]
	WP	Antibacterial	[68]
	WP, RT, L, FR	Antidote for snake bite, scorpion bite, bee bite, spider bite, blood clotting, leukoderma, skin infection, emollient, headache, leucorrhoea, dandruff, fever, constipation and abdominal colic, abortifacient, blood purifier, cholera, dryness of tongue, dysmenorrhea, watering of eye, gangrene, swelling in leg, stomach burning, pulmonary problems, arthritis, mastitis in animals, hemiplegia, anti-inflammatory, anti-oxidant, antidiabetic, larvicidal, antitumor	[22]
*A. krisagathra* Sivar. & Pradeep.	WP	Anti-inflammatory	[95]
	WP	Antiulcer	[119]
*A. kwangsiensis* Chun & F. C. How ex C. F. Liang.	LV	Antimicrobial, antioxidant, anti-inflammatory	[74]
*A. longa* L.	T	Antibacterial, cytotoxic, skin problems, gastrointestinal disorders	[23]
	S	Bronchitis, constipation, rheumatism, bladder diseases	[129]
	RT	Heart protector	[130]
	RT and AP	Antibacterial	[69]
	RT	Antioxidant	[80]
	RT and AP	Antibacterial, antioxidant	[70]
	RT	Antioxidant, antibacterial, cytotoxic	[25]
*A. macroura* Gomes.	LV	Cytotoxic	[32]
	AP	Antioxidant	[81]
*A. malmeana* Hoehne.	RT and LV	Insecticide	[105]
*A. maurorum* L.	RT and AP	Antiplatelet	[53]
*A. mollissima* Hance.	RZ and AP	Antibacterial,	[30]
	WP	Cytotoxic	[44]
*A. manshuriensis* Kom.	S	Anti-inflammatory	[93]
	LV	Antibacterial	[71]
*A. paucinervis* Pomel.	RT	Antiproliferative	[33]
*A. petersiana* Klotzsch.	RT	Antimalarial	[109]
*A. pubescens* Will. ex Duch.	RT and S	Insecticide	[106]
*A. odoratissima* L.	LV	Snake antivenom	[88]
	S	Antinociceptive	[111]
*A. orbicularis* Duch.	RT	Antibacterial	[72]
*A. ringens* Vahl.	RT	Cytotoxic	[31]
	SB	Antidiarrheal	[96]
	AP	Antibacterial, antifungal, cytotoxic	[28,45]
	RT	Antidiabetic	[82]
	RT	Antioxidant, antidyslipidemic	[82]
*A. saccata* Wall.	LV, RT, S, and T	Healing of wounds, body pain, diarrhea, dysentery, hemorrhage, jaundice, tonsil	[40,98]
*A. tadungensis* T. V. Do & Luu.	S and LV	Cytotoxic	[35]
*A. tagala* Cham.	RT and LV	Insecticide	[107]
	RT	Antioxidant, anti-inflammatory, anti-cancer	[46]
	RT	Anti-inflammatory, anti-cancer	[47]
	RT, LV, and WP	Stomach pain, chest pain, fever, poultice in abdomen, skin disease, snake bite, antimalarial, dyspepsia, flatulent, diarrhea, vomiting, headache, gynecological disorders, stimulate the menstrual flow, bone fracture, treatment of cancer	[48]
*A. taliscana* Hook. & Arn.	RZ	Antioxidant, antimicrobial	[52,55]
	RT	Antimycobacterial	[56,60]
*A. triangularis* Cham.	S	Antiproliferative, antibacterial	[49]
*A. trilobata* L.	LV	Acaricide	[131]
	S	Antinociceptive	[112]
	S	Antinociceptive, anti-inflammatory	[94]
*A. tuberosa* C. F. Liang & S. M. Hwang.	FT	Antinematode	[101]
*A. yunnanensis* Franch.	S	Antifibrosis	[115,116]
*A. zollingeriana* Miq.	FT and RT	Expectorant, antitussive, antihistamine, pain reliever, treatment of snake bites	[89]

^1^ AP = aerial parts, FT = fruits, LV = leaves, FR = fresh root, RT = roots, RZ = rhizomes, S = stems, SB = stem bark, T = tuber, WP = whole plant.

**Table 3:** IC_50_ values of crude extracts of the genus *Aristolochia*.

Cell Line	IC_50_ (mg/mL)	Species	Plant Part ^1^	Extract/Solvent ^2^	Reference
A431	0.0280	*A. ringens* Vahl.	RT	DCME:ME	[31]
A-549	0.0200	*A. ringens* Vahl.	RT	EE	[31]
	0.0260	*A. ringens* Vahl.	RT	DCME:ME	
	0.0755	*A. tadungensis* T. V. Do & Luu.	S and LV	ME	[35]
BSR	0.0600	*A. longa* L.	AP	DCM	[23]
	0.0180	*A. longa* L.	AP	HXE	
	0.3500 ^‡^	*A. longa* L.	AP	ME	
HBL-100	0.0400	*A. longa* L.	RT	H_2_O	[50]
HCT-116	0.0220	*A. ringens* Vahl.	RT	EE	[31]
	0.0195	*A. ringens* Vahl.	RT	DCME:ME	
HeLa	0.369 ^‡^	*A. galeata* Mart.	RZ	EE	[38]
	0.0300	*A. ringens* Vahl.	RT	DCME:ME	[31]
	0.0083	*A. tadungensis* T. V. Do & Luu.	S and LV	ME	[35]
Hep G-2	0.3800 ^‡^	*A. baetica* L.	RT	ME	[33]
	0.0164	*A. ringens* Vahl.	AP	CE	[28]
	0.5130 ^‡^	*A. macroura* Gomes.	LV	ME	[32]
HK-2	0.1826 ^‡^	*A. acumiata* Lam.	RT	ME	[137]
	>0.2000 ^‡^	*A. acuminata* Lam.	F		
	0.1574 ^‡^	*A. argentina* Griseb.	S		
	>0.2000 ^‡^	*A. baetica* L.	LV		
	>0.2000 ^‡^	*A. californica* Torr.	S		
	>0.2000 ^‡^	*A. chamissonis* Duch.	LV		
	0.0478	*A. clematitis* L.	SD		
	0.1633 ^‡^	*A. clematitis* L.	RT		
	>0.2000 ^‡^	*A. cymbifera* Mart.	S		
	>0.2000 ^‡^	*A. debilis* Siebold & Zucc.	S		
	>0.2000 ^‡^	*A. elegans* Mast.	LV		
	0.0911	*A. elegans* Mast.	RT		
	0.1881 ^‡^	*A. fangchi* Y.C. Wu ex L.D. Chow & S.M. Hwang.	S		
	0.1272 ^‡^	*A. grandiflora* Sw.	LV		
	>0.2000 ^‡^	*A. guentheri* O.C. Schmidt.	LV		
	0.0854	*A. guentheri* O.C. Schmidt.	S		
	0.1197 ^‡^	*A. labiata* Willd.	LV		
	>0.2000 ^‡^	*A. manshuriensis* Kom.	S		
	>0.2000 ^‡^	*A. maurorum* L.	LV		
	>0.2000 ^‡^	*A. maxima* Jacq.	RT		
	>0.2000 ^‡^	*A. odoratissima* L.	LV		
	>0.2000 ^‡^	*A. paucinervis* Pomel.	SD		
	0.1060 ^‡^	*A. ringens* Vahl.	RT		
	>0.2000 ^‡^	*A. rotunda* L.	RT		
	>0.2000 ^‡^	*A. tomentosa* Sims.	S		
	>0.2000 ^‡^	*A. trilobata* L.	LV		
	0.1424 ^‡^	*A. westlandii* Hemsl.	S		
	>0.2000 ^‡^	*A. zollingeriana* Miq.	LV		
HT-29	0.1000 ^‡^	*A. baetica* L.	RT	ME	[33]
MCF-7	0.2160 ^‡^	*A. baetica* L.	RT	CE	[26]
	0.0191	*A. bracteolata* Lam.	RT	EE	[34]
	0.3470 ^‡^	*A. indica* L.	LV	CE	[27]
	0.0816	*A. ringens* Vahl.	AP	CE	[28]
	0.0473	*A. foetida* Kunth	LV	DCME	[24]
	0.0459	*A. foetida* Kunth	S	DCME	
MDA-MB-231	0.0970	*A. longa* L.	RT	H_2_O	[50]
PANC-1	0.0826	*A. tadungensis* T. V. Do & Luu.	S and LV	ME	[35]
PC-3	0.0030	*A. ringens* Vahl.	RT	EE	[31]
	0.0120	*A. ringens* Vahl.	RT	DCME:ME	
RD	0.1254 ^‡^	*A. longa* L.	RT	DCME	[25]
	0.0625	*A. longa* L.	RT	ME	
	0.0151	*A. longa* L.	RT	HXE	
	0.0150	*A. longa* L.	AP	DCME	[23]
	0.2000 ^‡^	*A. longa* L.	AP	ME	
T-24	0.0480	*A. baetica* L.	RT	ME	[33]
THP-1	0.0240	*A. ringens* Vahl.	RT	EE	[31]
	0.0220	*A. ringens* Vahl.	RT	DCME:ME	
Vero	0.2500 ^‡^	*A. longa* L.	AP	DCME	[23]
	0.2500 ^‡^	*A. longa* L.	AP	HXE	
	0.0151	*A. longa* L.	RT	HXE	[25]
	0.0312	*A. longa* L.	RT	DCME	
	0.1253 ^‡^	*A. longa* L.	RT	ME	

^1^ AP = aerial parts, F = flower, LV = leaves, RT = roots, RZ = rhizomes, S = stems. ^2^ CE = chloroformic extract, DCME = dichloromethane extract, EE = ethanol extract, HXE = hexanic extract, ME = methanol extract. ^‡^ Shows concentrations > 0.1 mg/mL.
